# Characteristics of missing incidents and risk factors for harm for older people with and without dementia in the UK

**DOI:** 10.1093/ageing/afag130

**Published:** 2026-05-13

**Authors:** Vasiliki Orgeta, Garrett Kidd, Jean Stafford, Phuong Leung, Louise Marston, Aiden Sidebottom, Lawrence Fong

**Affiliations:** Division of Psychiatry, Faculty of Brain Sciences, University College London, London, UK; Division of Psychiatry, Faculty of Brain Sciences, University College London, London, UK; Advanced Care Research Centre, Usher Institute, College of Medicine and Veterinary Medicine, University of Edinburgh, Edinburgh, UK; Division of Psychiatry, Faculty of Brain Sciences, University College London, London, UK; Research Department of Primary Care and Population Health, University College London, London, UK; Department of Security and Crime Science, University College London, London, UK; Division of Psychiatry, Faculty of Brain Sciences, University College London, London, UK

**Keywords:** missing, dementia, older people, harm, risk, older people

## Abstract

**Background:**

Despite people with dementia being at increased risk of going missing, we currently have limited knowledge about the factors that increase their exposure to harm during missing incidents.

**Objective:**

To compare characteristics of missing incidents and associated risk factors for harm in older people with and without dementia reported missing in the UK.

**Methods:**

Retrospective analysis of reported missing incident data from two police forces in England and Wales over an 84-month period (January 2015 to December 2021).

**Results:**

A total of 1540 missing incidents involving 1286 people ≥65 years were identified, of which 414 (27%) incidents were dementia-related. In most incidents, people with dementia were reported missing from their own home/neighbourhood, with approximately half being found by the police. Nine percent (*n* = 39) sustained significant harm, including one death. Older age, going missing at night and prolonged duration of going missing were associated with increased probability of harm. Approximately 14% of people with dementia and 11% of people without dementia experienced at least one repeat missing incident.

**Conclusions:**

Our study is the largest to date reporting on missing incidents among older people in the UK. Despite being a low frequency event, missing incidents are associated with significant risk of harm for older people with and without dementia. Preventative and timely interventions to prevent harm associated with missing incidents for those aged over 65 years with or without dementia are urgently needed.

## Key Points

- Our understanding of the risk factors for harm of dementia-related missing incidents remains limited.
- We analysed police-recorded missing incidents of older people with and without dementia over an 84-month period in the UK.
- Older age, missing at night and missing for more than 24 hours were associated with increased probability of harm.
- Fourteen percent of people with dementia went missing more than once.
- Effective preventative strategies for missing incidents in older people with and without dementia are urgently needed

## Introduction

Dementia represents a major public health concern [1], affecting more than 57 million people globally [2]. Participating in outdoor activities is an important aspect of living well with dementia [3], yet for many people with dementia, walking and engaging in outdoor activities can increase risk of going missing [4]. Missing episodes can result in significant harm, such as injury and death [5, 6], and are distressing events for both the person with dementia and family carers [7]. In the UK alone, missing person investigations are estimated to cost over £700 million annually, with approximately a third of this cost attributed to dementia-related incidents [8, 9]. This highlights a broader global concern, as ageing populations worldwide are likely to face similar challenges and associated economic burdens.

Despite projections indicating a global rise in dementia-related missing incidents [6], effective interventions to prevent harm during such episodes are currently lacking across most countries [10, 11], highlighting a significant gap in prevention and care. This often results in the use of ‘restrictive strategies’ (e.g. setting rigid routines, limiting choices, imposing constant supervision) after a missing episode by both family and paid carers, limiting participation in outdoor activities [12, 13], which can contribute to poorer outcomes, including entering long-term care prematurely [14]. Understanding the characteristics of missing incidents and risk factors for harm is therefore crucial for developing interventions to prevent harm [11].

Robust global estimates of the prevalence of dementia-related missing episodes are currently unavailable [15], partly due to the different definitions used [16, 17], and variations in use of carer-reported versus police-recorded estimates [13, 18]. However, despite these limitations, studies indicate that the prevalence of missing episodes among people with dementia could be as high as 40% [18–20].

In the UK, reported missing incidents are primarily managed by the police, who follow national guidelines and risk assessment frameworks to coordinate search and safeguarding efforts [21]. While some dementia-related missing incidents are resolved quickly, a significant proportion of individuals with dementia who go missing may experience serious injury [6, 22]. International evidence from Japan and the United States suggests that the duration of a missing episode and living alone are key predictors of mortality risk [5, 23, 24], with those not found within 24 hours more likely to experience severe harm [25].

Although data on dementia-related missing incidents in the UK remain limited, the few studies conducted so far indicate that around 5% of these cases result in severe harm [22]. Additionally, there has been limited investigation of missing incidents using UK police data, creating a significant gap in our understanding of these incidents and the factors that predict exposure to harm [26].

Therefore, an important aim of the present study was to examine the characteristics, harms and risk factors for harm associated with dementia-related missing incidents, drawing on a large multi-year police-recorded missing incidents dataset. Given recent estimates that individuals aged ≥65 years are more likely to experience severe harm during missing episodes compared to those aged <65 years [21], we also examined missing incidents involving older adults without recorded dementia.

## Method

### Data

We analysed data of missing person incidents recorded by two police forces in England and Wales during January 2015 to December 2021, inclusive [21]. These comprised information on the persons’ age, sex and presence of dementia diagnosis. Missing incident details recorded included: (i) time, (ii) date, (iii) location of the missing incident and (iv) who found the missing person. Additional variables were: (i) number of hours a person was reported missing, (ii) number of missing incidents per person over the 84-month study period, (iii) distance between location found and place recorded missing from and (iv) whether the missing incident resulted in harmful outcomes (unharmed/harmed but alive/deceased). Dementia diagnosis was based on caregiver reports rather than clinical records. The original dataset comprised 44 313 missing incidents involving 19 873 individuals (for further details of this dataset, see [21]). Our analytic sample focused on people aged ≥65 years.

### Data analysis

All variables were described and analysed at the incident level except sex and whether individuals went missing more than once, which were described at the person level. To explore possible risk factors associated with harm, univariable logistic regressions were first conducted on each potential predictor variable (stratified by the presence or absence of dementia), to obtain baseline associations between predictors and presence of harm (yes/no). For individuals who went missing more than once, only their first incident was included in the regression analyses; repeat incidents were excluded. Then, two multivariable logistic regressions were conducted for people with and without dementia. Predictor variables were chosen based on their significance in univariable models and the 10 events per variable rule. Distance travelled while missing was excluded from multivariable regressions regardless of statistical significance (data were unavailable for approximately half of the total incidents). If the chosen predictor variables contained missing/unknown data, the corresponding incidents were excluded. Outcomes were presented as odds ratios (ORs) alongside 95% confidence intervals (CIs). Incidents with unknown harm data were excluded from all regression analyses.

Categories with zero observations for people with or without dementia were dropped from regression analyses. All assumptions for logistic regression were met, and no multicollinearity was detected (all variance inflation factor values <1.05). For all statistical tests, statistical significance was set at *P* < .05 with all analyses performed using the Statistical Package for the Social Sciences (SPSS) for Windows (version 29).

## Results

### Missing incident characteristics and differences for people over 65 with and without dementia

Our analytic sample consisted of 1540 incidents (3.5% of all police-recorded missing incidents over the study period). Table 1 shows descriptive statistics for missing incidents involving older people with and without dementia. Of the 1540 incidents involving 1286 older people, 414 incidents (339 people) were dementia-related, which accounted for 26.9% of all incidents involving older people. The median ages in incidents of people with and without dementia reported missing were 80 (IQR 75–84) and 73 (IQR 68–80) years, respectively, and 45.7% of individuals with dementia reported missing were female, which was higher than the 31.5% females in missing people without dementia.

**Table 1:** Descriptive statistics for missing incidents involving older people aged ≥65 with and without dementia.

Incidents (*n* = 1540)	With dementia (*n* = 414)	Without dementia (*n* = 1126)
	*N* (%)	*N* (%)
Age, median (IQR)	80 (75–84)	73 (68–80)
*Sex* ^a^		
Female	155 (45.7)	298 (31.5)
Male	184 (54.3)	648 (68.4)
Unknown	–	1 (0.1)
*Place reported missing*		
Home/neighbourhood	321 (77.5)	941 (83.6)
Residential care	57 (13.8)	44 (3.9)
Leisure facility/public transport/street or other public place	20 (4.8)	34 (3.0)
Hospital	16 (3.9)	107 (9.5)
*Who found the person*		
Police	192 (46.3)	614 (54.5)
Family/friends/acquaintance	55 (13.3)	132 (11.7)
Returned on their own	36 (8.7)	119 (10.6)
Care home staff/health services staff	40 (9.7)	61 (5.4)
Other/members of the public/other agency	88 (21.3)	177 (15.7)
Unknown	3 (0.7)	23 (2.1)
*Time of day reported missing*		
Day (8 a.m.–8 p.m.)	332 (80.2)	804 (71.4)
Night (8 p.m.–8 a.m.)	82 (19.8)	322 (28.6)
*Time of year reported missing*		
May–October (warmer months)	229 (55.3)	638 (56.7)
November–April (cooler months)	185 (44.7)	488 (43.3)
*Duration from reported missing to being found*		
Fewer than 24 hours	402 (97.1)	1009 (89.6)
More than 24 hours	12 (2.9)	117 (10.4)
*Went missing more than once* ^a^		
Single time only	290 (85.5)	841 (88.8)
Repeat missing	49 (14.5)	106 (11.2)
*Distance travelled while missing*		
0–5 miles	179 (43.2)	469 (41.7)
Over 5 miles	18 (4.4)	134 (11.9)
Unknown	217 (52.4)	523 (46.4)
*Harm experienced*		
Unharmed	373 (90.1)	1003 (89.1)
Sustained harm (alive)	38 (9.2)	80 (7.1)
Dead	1 (0.2)	38 (3.4)
Unknown	2 (0.5)	5 (0.4)

^a^ *n* = 1286 unique individuals

People with dementia were mostly reported missing from domestic settings (77.5%), followed by care homes (13.8%), public locations (4.8%) and hospitals (3.9%); people without dementia were also mainly missing from domestic settings (83.6%), but more commonly from hospitals (9.5%) and less from care homes (3.9%) than people with dementia. Nearly half (46.3%) of people with dementia were found by the police, followed by members of the public (21.3%), family or friends (13.3%), care home or health services staff (9.7%) or returning home on their own (8.7%). Missing people without dementia largely mirrored this distribution, with 54.5% of them found by the police, except they were less likely to be found by care home or health services staff (5.4%).

A higher proportion of people with dementia were reported missing during daytime hours (80.2%) than people without dementia (71.4%). More people went missing during warmer months than cooler months, for both people with (55.3%) and without dementia (56.7%). People with dementia were more likely to be missing for fewer than 24 hours (97.1%) compared to those without (89.6%). Among known cases, 9.1% of people with dementia travelled >5 miles while missing, a much lower proportion compared to 22.2% in people without dementia.

### Repeat missing incidents

Of the 414 dementia-related missing incidents, 290 were single incidents. The remaining 124 incidents involved 49 unique individuals who went missing repeatedly within the study period. Of these repeatedly missing persons, 34 of them were recorded missing twice, 9 were missing 3 times and 6 were missing 4 or more times. Approximately 85.5% of individuals with dementia went missing once, with 14.5% repeatedly going missing.

As for the 1126 non-dementia-related missing incidents, 841 were single incidents. The remaining 285 incidents involved 106 unique individuals who went missing repeatedly. Seventy-five were missing twice, 15 were missing 3 times and 16 were missing 4 or more times, and 88.8% of individuals without dementia went missing only once; 11.2% repeatedly went missing.

### Experience of harm

A total of 39 (9.4%) dementia-related missing incidents resulted in significant harm, which included experiencing accidents, physical injury, self-harm and one death (0.2%), whereas 118 (10.5%) incidents relating to people without dementia led to harm, including 38 deaths (3.4%).

### Factors associated with harm

Table 2 shows the factors associated with the occurrence of harm in missing episodes for both groups. Univariable logistic regressions showed that for people with dementia, age (being older), who located the missing person (care home or health services staff, and members of the public compared to the police), time of day reported missing (nighttime) and duration of missing incident (>24 hours) were significantly associated with increased probability of harm. In the multivariable logistic regression model, with predictors being age, time of day reported missing and duration of missing incident, all factors remained statistically significant. The odds of harm were 1.12 times higher with each additional year of age (95% CI 1.06–1.20). Being reported missing at night (OR = 2.94, 95% CI 1.34–6.48) and for more than 24 hours (OR = 9.91, 95% CI 2.20–44.68) were also associated with increased probability of harm.

**Table 2:** Factors associated with harm in missing incidents involving older people aged ≥65 with and without dementia.

First incidents with data on harm (*n* = 1281)	With dementia (*n* = 337)				Without dementia (*n* = 944)			
	Univariable logistic regression		Multivariable logistic regression (*n* = 412)		Univariable logistic regression		Multivariable logistic regression (*n* = 1103)	
	OR	95% CI	OR	95% CI	OR	95% CI	OR	95% CI
Age	1.12^**^	1.05–1.18	1.12^**^	1.06–1.20	1.00	0.97–1.03	–	–
*Sex*								
Female	Ref	–	–	–	Ref	–	–	–
Male	1.39	0.68–2.81	–	–	1.01	0.65–1.55	–	–
*Place reported missing*								
Home/neighbourhood	Ref	–	–	–	Ref	–	–	–
Residential care	1.24	0.48–3.19	–	–	0.63	0.19–2.08	–	–
Leisure facility/public transport/street or other public place	0.48	0.06–3.70	–	–	0.48	0.11–2.03	–	–
Hospital	–	–	–	–	0.25^*^	0.08–0.79	–	–
*Who found the person*								
Police	Ref	–	–	–	Ref	–	Ref	–
Family/friends/acquaintance	0.93	0.25–3.53	–	–	0.66	0.30–1.42	0.66	0.30–1.44
Returned on their own	–	–	–	–	0.29^*^	0.09–0.93	0.28^*^	0.09–0.93
Care home staff/health services staff	3.45^*^	1.21–9.82	–	–	1.85	0.82–4.18	2.01	0.88–4.56
Other/members of the public/other agency	4.24^**^	1.81–9.91	–	–	3.64^**^	2.28–5.81	3.43^**^	2.14–5.51
*Time of day reported missing*								
Day (8 a.m.–8 p.m.)	Ref	–	Ref	–	Ref	–	–	–
Night (8 p.m.–8 a.m.)	2.65^*^	1.26–5.55	2.94^**^	1.34–6.48	1.37	0.89–2.09	–	–
*Time of year reported missing*								
May–October (warmer months)	Ref	–	–	–	Ref	–	Ref	–
November–April (cooler months)	0.73	0.36–1.49	–	–	1.51^*^	1.01–2.26	1.46	0.96–2.22
*Duration from reported missing to being found*								
Fewer than 24 hours	Ref	–	Ref	–	Ref	–	Ref	–
More than 24 hours	9.28^**^	2.21–38.90	9.91^**^	2.20–44.68	2.60^**^	1.54–4.39	2.18^**^	1.25–3.81
*Went missing more than once*								
Single time only	Ref	–	–	–	Ref	–	–	–
Repeat missing	0.33	0.08–1.40	**–**	–	0.62	0.29–1.31	–	–
*Distance travelled while missing*								
0–5 miles	Ref	–	–	–	Ref	–	–	–
Over 5 miles	3.62	0.86–15.21	–	–	0.95	0.47–1.92	–	–

^*^ *P* < .05; ^**^P < .01.

As for older people without dementia, univariable logistic regressions showed that duration of missing incident (>24 hours) and time of year reported missing (during cooler months) were significantly associated with higher probability of harm. Compared to being found by the police, being found by members of the public was associated with greater probability of harm, whereas returning home of their own accord was associated with lower probability of harm. Factors associated with harm in univariable models remained associated in the multivariable regression model except for time of year reported missing. Being missing for more than 24 hours was associated with double the odds of harm (OR = 2.18, 95% CI 1.25–3.81). Compared to being found by the police, being found by members of the public (OR = 3.43, 95% CI 2.14–5.51) was associated with increased probability of harm. However, returning home of their own accord (OR = 0.28, 95% CI 0.09–0.93) was associated with decreased odds of harm.

## Discussion

This study is the largest to date reporting on characteristics, exposure to and risk factors for harm associated with missing episodes for people with dementia in the UK. To our knowledge, this is the first UK study to examine differences in police-recorded missing incidents between older people with and without dementia using a large multi-year dataset. As predicted by previous studies [22, 26, 27], we found that missing episodes were most common in residential settings compared to long-term care or hospitals. While these findings highlight the potential value of future research focusing on the development and evaluation of preventative interventions in domestic settings [22], it is equally important not to overlook interventions in care homes and hospitals.

Consistent with findings from other countries, almost half of incidents of people with dementia reported missing were found by the police and one-fifth by members of the public [26, 27]. The finding that a large proportion of people with dementia are located and brought to safety by the public has significant implications for future prevention efforts, suggesting that public awareness interventions targeting members of the public [27] are likely to be key in improving our wider public health response to missing incidents in dementia [12].

Our study also found that people with dementia who were reported missing were more likely to be female and older than those without dementia. However, these associations may partly reflect the role of age and sex as primary risk factors for dementia itself, rather than as independent predictors of going missing, which limits their practical and policy significance. Future research is needed to disentangle dementia-related risk from demographic confounding to better identify modifiable factors for prevention.

Four in five people with dementia were reported missing during daytime hours, with the vast majority (97%) missing for fewer than 24 hours. These results suggest that most family carers report a missing incident promptly [22, 28]. However, given that time missing was dichotomised (<24 hours vs. ≥24 hours), our findings provide limited insight into optimal reporting windows for people with dementia. Despite this limitation, however, we found that people with dementia, on average, travelled shorter distances during a missing episode than people without dementia—an observation that aligns with previous international evidence from the United States [25, 29].

An important finding of our study is that repeat missing incidents were observed in up to 15% of people with dementia. While repeat incidents may indicate opportunities for targeted preventative interventions [11], it is important to note that these findings may not be generalisable to the broader population. Future research should investigate repeat missing incidents using more representative datasets involving general population samples to better understand their prevalence and implications for targeted interventions.

We examined harm associated with police-recorded missing incidents, including cases resulting in death. The proportion of people with dementia experiencing physical injury in our study (9%) is consistent with some estimates in the literature [27, 30], but higher than those reported in population-based studies [28]. This likely reflects the nature of police-reported data, which may disproportionately capture more severe cases, rather than representing the full spectrum of missing episodes. Our data suggested that people with dementia were less likely to experience mortality than people without dementia. Nonetheless, given that some individuals with dementia do experience serious injuries or death, interventions aimed at preventing or reducing harm remain essential and should be guided by population-level evidence [27, 28].

We found that increasing age was associated with higher odds of harm in people with dementia, and that individuals who experienced harm were more likely to be found by care home staff or members of the public. This likely reflects the circumstances in which harm occurs rather than suggesting that being found by a particular group influences risk. Timely and coordinated interventions by search and rescue teams and the police therefore remain important, and evaluating the effectiveness of these interventions should be a priority for future research [11]. Being reported missing at night and being missing for more than 24 hours were also associated with increased probability of experiencing harm. These factors are in line with previous studies [22, 26] and highlight the need for timely interventions, particularly if people are reported missing at night, which increases the probability of severe harm including death [18, 31].

Despite its strengths, this study has several limitations. First, not all missing incidents are reported to the police, and reporting practices vary across regions, leading to underestimation. Biases may also exist in who is reported missing (e.g. by socioeconomic status, ethnicity, age, sex or region), which could skew results. Second, measures of harm were derived from police records rather than clinical data, meaning some harms may have been under- or over-reported. Similarly, classification of dementia-related incidents relied on caregiver reports rather than formal diagnoses. As a result, some cases may have been misclassified—for instance, older adults recorded as not having dementia may have had undiagnosed dementia or may have gone missing for other reasons (e.g. social or mental health difficulties). This misclassification could partly explain the high number of incidents among people over 65.

Third, reliance on police-reported cases likely captured only the most severe missing incidents, excluding those resolved informally, which may bias risk factor profiles and outcomes. The comparison group comprised older adults without dementia who also went missing, as police records do not capture individuals with dementia who have never gone missing. This limited the ability to identify dementia-specific risk factors. Finally, police records may not be fully representative of the wider UK population, constraining the generalisability of findings.

Accurate and detailed reporting of missing incidents, including the nature of harm experienced, is a critical research priority worldwide [5, 23, 32]. Future large-scale prospective studies that compare the incidence of missing episodes across different countries—between carer-reported and police-recorded incidents—are urgently needed to better understand variations in reporting and response. While our study assessed physical harm, it did not address psychological harm experienced by carers [7], or the impact of placement in long-term care, both of which carry significant social and economic costs globally for individuals, families and societies [33].

Despite these limitations, our study provides important new information for the global study of missing incidents in dementia. A focus on predictors of repeat missing episodes may aid the development of assessment protocols to ensure risk of future missing episodes can be reduced. Future studies should also include younger people affected by early-onset dementia. Psychoeducation and awareness about missing incidents for families [34], and specific guidelines about when to report a person with dementia going missing [11, 35], will be key in preventing the risk of harm [9]. Given wide ethnic disparities in many areas of dementia health care, future studies should investigate missing incidents and outcomes for people from ethnic minority groups [36].

## Conclusion

The development of effective preventative and response strategies for missing incidents in older people with and without dementia is urgently needed. These strategies should focus on reducing and preventing adverse consequences associated with missing incidents as well as preventing occurrence of future episodes. With the global population of older adults and people affected by dementia rapidly increasing, interventions that support individuals in maintaining an active life within their communities and minimise harm associated with missing episodes should be integral components of health and social care systems worldwide.
